# Cognitive Behavioral Therapy (CBT) in Psychogenic Non-Epileptic Seizures (PNES): A Case Report and Literature Review

**DOI:** 10.3390/bs9020015

**Published:** 2019-01-29

**Authors:** Saher Hoda Kamil, Mustafa Qureshi, Rikinkumar S. Patel

**Affiliations:** 1 Department of Psychiatry, Austin State Hospital, Austin, TX 78751, USA; saherumair@gmail.com; 2 Department of Psychiatry, Texas Tech University Health Science Center, Midland, TX 79701, USA; dr.qureshi.mustafa@gmail.com; 3 Department of Psychiatry, Griffin Memorial Hospital, Norman, OK 73071, USA

**Keywords:** PNES, psychogenic non-epileptic seizures, pseudoseizures, CBT, cognitive behavioral therapy, management, inpatient psychiatry, EEG

## Abstract

Psychogenic non-epileptic seizures (PNES) are classified as a somatoform conversion disorder. We present a case of a 24-year-old male with a past psychiatric history of posttraumatic stress disorder (PTSD) and anxiety disorder, admitted to our inpatient psychiatric unit. The patient experienced multiple episodes of seizures during hospitalization. Work up was unremarkable, and PNES were suspected and later confirmed with video-electroencephalography (video-EEG). He underwent supervised withdrawal of antiepileptic medications with the initiation of cognitive behavioral therapy (CBT), which reduced the frequency of seizures. Diagnosis of PNES can present as a challenge and failure to diagnose its psychological nature can lead to a delay in the psychological intervention. CBT leads to a decrease in seizure frequency, and improvement in psychiatric symptoms, psychosocial functioning, and quality of life. It is important to consider PNES in the differential diagnosis of seizures presented by psychiatric patients, as CBT is necessary for better patient outcomes.

## 1. Introduction

Psychogenic non-epileptic seizures (PNES) are a somatoform conversion disorder of psychological origin, presenting as paroxysmal episodes with normal electroencephalographic (EEG) findings [1]. Patients who present with PNES have maladaptive coping strategies when they are under stress [2,3]. These seizures are unintentional and are psychological defense mechanisms against internal stressors [4]. The prevalence of PNES is two to thirty-three per 100,000 individuals [5]. About 75–85% of those diagnosed with PNES are females with the beginning of seizures in early adulthood [6].

The most common comorbid psychiatric diagnoses seen in patients with PNES are depression, post-traumatic stress disorder (PTSD), and panic disorder [4]. This is similar to patients with epilepsy, who often present with comorbid depression [7]. Many patients also have a history of sexual and/or physical abuse, with traumatic experiences producing dissociation [4,8]. This is further supported by Myers et al. [9], who concluded that individuals with PNES are exposed more to psychological trauma as compared to the general population.

About 10–20% patients presenting to epilepsy centers have PNES [5]. Symptoms of PNES have a psychiatric origin and neurologic presentation [10]. It is hard to differentiate PNES from epilepsy, and many patients are treated with anticonvulsants for several years before a correct diagnosis is made [11]. There is a delay of about seven years between manifestation and diagnosis of PNES [12]. Such delay in proper diagnoses and treatment results in significant morbidity due to adverse side effects of antiepileptic medications [13]. Early diagnosis and management of PNES are cost-effective with about an 84% decrease in total medical charges. Confirmation of PNES with 24-h video-EEG helps reduce healthcare utilization and cost [14].

The treatment of PNES remains controversial, with psychological and pharmacological interventions mentioned in literature. Pharmacological interventions focus on antidepressants and anxiolytics, and psychological interventions include cognitive behavioral therapy (CBT), psychodynamic therapy, cognitive analytical therapy, interpersonal therapy, family therapy, hypnotherapy, and paradoxical therapy [15]. A meta-analysis of thirteen studies that included 228 individuals with PNES demonstrated the effect of psychological interventions on seizure frequency, including CBT, psychodynamic therapy, paradoxical intention therapy, mindfulness, and psychoeducation with eclectic interventions. There was cessation of seizures in 47% of the individuals upon completion of the therapy [16]. There have been many studies conducted in the past that have recognized the benefit of CBT for managing PNES [17,18,19], and a study conducted by Goldstein et al. [20]. provided the strongest evidence for CBT effectiveness.

Herein, we will describe a patient admitted to the inpatient unit at a state hospital for homicidal ideations, who was eventually diagnosed with PNES during psychiatric management. We will also explore the usefulness of CBT in reducing seizure frequency after reviewing past studies published in the MEDLINE database.

## 2. Method

A literature search on MEDLINE was conducted from January 1, 2005 to November 1, 2018 using the keyword “PNES”, “psychogenic non-epileptic seizures”, or “pseudoseizures”, and cross-referencing it with either “CBT” or “cognitive behavioral therapy”. Articles found through this indexed search were reviewed and screened to include relevant original research studies, including randomized case-control studies and prospective interventional studies, to evaluate the efficacy and outcomes of CBT in patients with PNES.

Ethics and Human Subject: Case that is discussed in this article was only mentioned after receiving a verbal consent from the patient.

## 3. Case Presentation

A 24-year-old African American male with a past psychiatric history of PTSD and anxiety, and multiple past psychiatric hospitalizations, was admitted to the adult inpatient psychiatric unit for homicidal ideation, with intent or plan. On day one, the patient was calm and cooperative and denied depression, suicidal or homicidal ideation, or auditory or visual hallucinations. He reported being sexually, physically, and emotionally abused by his mother’s live-in boyfriend at the age of eight. He had a past medical history of seizures and was on phenytoin 400 mg twice daily and valproic acid 2000 mg twice daily.

During his hospital course, the patient experienced multiple episodes of seizures, with a cluster event of five seizures in 20 min on the third day. The patient’s valproic acid dosage was titrated to 3000 mg twice daily. He remained in a postictal state for a brief period following the seizures, with urinary incontinence. Lorazepam 2 mg intramuscular was administered stat (immediately) with each episode, and the patient was placed on every 15 min check (Q15) and one-to-one observation for seizure monitoring. Emergency medical services (EMS) were called multiple times, and work-up of complete blood count (CBC), prolactin level, thyroid-stimulating hormone (TSH) level, and complete metabolic panel (CMP) revealed results within normal ranges. EEG showed no focal slowing and no epileptic activity. The resting background rhythm was normal, and the patient was suspected of having PNES. The 24-h video-EEG did not show any abnormal activity during the paroxysmal events, confirming the seizures were non-epileptic.

It was recommended to undergo supervised withdrawal of antiepileptic drugs and start CBT, as the seizure-like activity was thought to be psychological in origin. The etiology and treatment approach for PNES were carefully explained to the patient. CBT was initiated, with one session per week for ten weeks. The therapy primarily focused on trauma and aggression. It was discussed how the patient’s thoughts impacted his emotions, behavior, and decision-making. He was encouraged to monitor his thoughts and feelings and how they influenced each other. Coping skills that can be used when the patient experiences nightmares and flashbacks were also discussed. The patient was encouraged to process his thoughts and feelings through story-writing, journaling, and music. Thinking errors were further reviewed, and the patient’s tendency to assume what others were thinking and feeling were discussed, with the aim of helping the patient deal with problematic events in a more effective manner. As the CBT sessions continued, the patient’s frequency of seizures gradually reduced from seven times per week to four times per week mid-way through therapy sessions, and finally zero times weekly at the completion of the ten therapy sessions. With the improvement in PNES symptomatology as well as patient’s insight and judgment, he was discharged with a scheduled outpatient neurology follow-up.

## 4. Discussion and Review of Literature

A diagnosis of PNES can only be made with a thorough and detailed history, confirmed with 24-h video-EEG [1]. The patient can get frustrated when there is no medical explanation for the seizure, and hence the physician should be careful in explaining PNES, as it is likely for the patient to reject the diagnosis and decline treatment options [21]. It is not uncommon for patients with PNES to undergo unnecessary diagnostic tests and treatments, with effective therapies being delayed [22]. The next step is to taper antiepileptic medications and titrate psychotropic drugs for the treatment of psychiatric comorbidities, if present [1]. Psychotherapy is the treatment of choice as PNES do not respond to pharmacotherapeutic treatment and can actually be worsened with the use of anticonvulsants [23,24]. Completion of psychological interventions like CBT, psychodynamic therapy, mindfulness therapy, and psychoeducation, can lead to cessation or reduction of seizures, as demonstrated by Carlson et al. [16], and among these, CBT holds the most reliable evidence of efficacy.

Some studies have demonstrated the effectiveness of CBT in the treatment of PNES. LaFrance et al. [25] demonstrated a decrease in seizure frequency, and improvement in psychiatric symptoms, psychosocial functioning, and quality of life, in patients receiving CBT over twelve weekly sessions. In this study, 16 out of 21 patients experienced a 50% reduction in seizures, and 11 out of 17 were seizure free at the end of treatment, with seizure frequency reduced from a median of eight per week to zero per week. Later, LaFrance et al. [26] conducted a multi-center pilot randomized controlled trial (RCT) to compare CBT-informed psychotherapy (CBT-ip), CBT-ip with sertraline, sertraline alone, and treatment as usual (TAU) in patients with PNES. This study resulted in seizure reduction of up to 51.4% and 59.3% in CBT-ip and CBT-ip with sertraline groups, respectively, as compared to 26.5% in sertraline only and 33.8% in TAU groups. The CBT-ip groups (without and with sertraline) also resulted in a reduction of depression and anxiety symptoms, with improved quality of life and global functioning [26]. This study was further elaborated by Baird et al. [27], showing a reduction in the cluster events in the CBT-ip groups. Cluster events are when two to four seizures occur within 48 h [28]. Hence, it can be concluded that CBT-ip not only reduces seizure frequency but also cluster events [27].

A pilot study conducted by Goldstein et al. [29] showed the effectiveness of CBT on seizure frequency and psychosocial functioning, including employment status and mood, before and at the end of treatment, as well as at a six-month follow-up. This study resulted in a reduction in seizure frequency and improvement in psychosocial functioning at the completion of twelve CBT sessions, with the trend being maintained at the six-month follow-up [29]. A few years later, Goldstein et al. [20] conducted another pilot RCT over four months to compare CBT combined with standard medical care (SMC) to SMC alone in patients with PNES. The study resulted in reduced PNES frequency at the end of treatment, with the trend being maintained at the six-month follow-up, and an absence of PNES for three consecutive months following CBT. Monthly seizure frequency reduced from a median of 12 per month at the start of treatment to two per month at the end of treatment, along with 1.5 per month at the six-month follow-up in the CBT group. In the SMC group alone, the seizure frequency reduced from eight at the start of treatment to 6.75 by the end of treatment, with five seizures per month at the follow-up [20].

A study conducted by Cope et al. [30] demonstrated PNES patients being managed with three 90-min group CBT-based psychoeducation sessions. These sessions were designed to help patients understand their diagnosis, meet others with the same diagnosis, recognize unhealthy thoughts, and learn to manage their symptoms. Forty percent of patients became seizure-free by the end of the group treatment, as compared to 11% at the beginning of the treatment. There were also significant improvements in psychological distress levels and understanding of illness [30]. A study by de Barros et al. [31] also supports the usefulness of CBT in patients with PNES and comorbid refractory medial temporal lobe epilepsy. Patients were placed in eight weekly semi-structured group sessions of psychological intervention based on CBT. CBT resulted in decreased frequency of seizures, reduced levels of depression, anxiety, and alexithymia, and improved quality of life.^31^ There was one study that showed the efficacy of CBT in the pediatric population diagnosed with PNES. Patients treated with CBT (up to 14 sessions) had full or partial remission of seizures in 79% of the cases over few months, along with a sevenfold decrease in emergency department (ED) visits [32].

It is important to diagnose and treat PNES as early as possible to reduce healthcare costs and improve a patient’s quality of life. It is also important to recognize psychiatric comorbidities and treat them as part of the work-up in treatment of PNES. A study has shown the influential effects of psychiatric comorbidities with epilepsy on health-related quality of life, and highlighted the importance of diagnosis and treatment of comorbid psychopathology in epileptic patients [7].

## 5. Conclusions

According to our experience with our patient, early diagnosis and management of PNES is crucial and should be focused on CBT, as PNES involves more distress due to adverse life events. When seizure management becomes difficult, then video-EEG should be conducted to include or exclude PNES. This will eliminate unnecessary diagnostic tests and use of antiepileptic medications, and ultimately not only improve the remission rate but also reduce healthcare utilization and cost. Psychotherapeutic management of the comorbid psychiatric condition is also required to improve the long-term patient outcomes, including psychosocial functioning and quality of life.
